# miR-19a acts as an oncogenic microRNA and is up-regulated in bladder cancer

**DOI:** 10.1186/s13046-014-0067-8

**Published:** 2014-08-10

**Authors:** Yougang Feng, Jun Liu, Yongming Kang, Yue He, Bo Liang, Ping Yang, Zhou Yu

**Affiliations:** 1 Department of Urology, Suining Central Hospital, 127 Deshengxi Road, Chuanshan District, Suining 629000, P R China

**Keywords:** Bladder cancer, miR-19a, PTEN, Circulation miRNA

## Abstract

**Background:**

The application of microRNAs (miRNAs) as potential biomarkers and therapy targets has been widely investigated in many kinds of cancers. The discovery of tumor associated miRNAs in serum of patients supported the use of plasma/serum miRNAs as noninvasive means of cancer detection. However, the aberrant expression of miRNAs in bladder cancer patients and their intensive roles and mechanisms in bladder cancer are poorly understood.

**Methods:**

Taqman probe stem-loop real-time PCR was used to accurately measure the levels of miR-19a in bladder cancer cell lines, 100 pairs of bladder cancer tissues and the adjacent non-neoplastic tissues and also the plasma collected from bladder cancer patients and normal controls. miR-19a mimics and inhibitors were transfected into bladder cancer cells to investigate its role on regulating cell proliferation which was measured by CCK-8 and colony formation assay. The target of miR-19a was identified by western blot and whether its regulatory role depends on its target was improved by a rescue experiment with miR-19a mimic and PTEN expression plasmid.

**Results:**

miR-19a was significantly up-regulated in bladder cancer tissues and high-level of miR-19a was correlative with more aggressive phenotypes of bladder cancer. Meanwhile, gain or loss of function of miR-19a demonstrated that miR-19a can promote cell growth of bladder cancer cells and the further mechanism studies indicated that its oncogenic role was dependent on targeting PTEN. Furthermore, investigation of miR-19a expression in the plasma of bladder cancer patients showed that miR-19a was also increased in plasma of bladder cancer patients which strongly supported miR-19a could be developed as potential diagnostic marker of bladder cancer.

**Conclusions:**

Our data indicated that miR-19a might act as an oncogenic microRNA in bladder cancer and was significantly up-regulated in bladder cancer carcinogenesis. The oncogenic role of miR19a in bladder cancer was dependent on targeting PTEN.

## Background

Bladder cancer is one of the most frequent malignancies in the world which includes several types of malignancy arising from the epithelial lining of the urinary bladder. Chromosomal anomalies, genetic polymorphisms, genetic and epigenetic alterations have been reported to be included in the tumorigenesis and progression of bladder cancer [1]. Recently, the applications of microRNAs (miRNAs) as potential biomarkers and therapy targets have been widely investigated in many kinds of cancers.

MiRNAs are endogenous small non-coding RNA molecules functioning in transcriptional and post-transcriptional regulation of gene expression. Recent studies have documented that miRNAs act as oncogenes or tumor suppressors in a variety types of cancer, such as lung, breast, hepatic, and pancreatic cancer [2]–[7]. Currently, the aberrant expression of miRNAs has been observed in bladder cancer and several miRNAs have been reported to play important roles in bladder cancer tumorigenesis and progression. For example, miR-582-5p and miR-582-3p are decreased in high-grade bladder cancer clinical samples, and synthetic miR-582 molecule can suppress bladder tumor growth and metastasis in animal model [8]. miR-125b was reported to suppress bladder cancer development by down-regulating oncogene SIRT7 and oncogenic long noncoding RNA MALAT1 [9]. Down-regulation of miR-99a/100 in bladder cancer tissues and their tumor suppressor roles in bladder cancer cells was also reported [10]. In addition, some preliminary experiments suggested that miR-23b, miR-16, miR-124-3p and miR-26a might function as tumor suppressors in bladder cancer [11]–[14]. Meanwhile, miR-21 was reported to be up-regulated in high-grade bladder cancer and can suppress p53 function [10]. Several oncogenic miRNAs including miR-144, miR-10b, miR-200c and so on were reported to be involved in bladder cancer progression [15],[16]. However, the aberrant expression of miRNAs in numbers of bladder cancer patients and their intensive roles and mechanisms in bladder cancer are poorly understood.

miR-19a/b are recognized to be the most important miRNAs in the oncomiRs—miR-17-92 cluster. miR-19a/b has been reported to be deregulated in many kinds of cancers including acute myeloid leukemia, colorectal cancer and gastric cancer, and might promote tumor growth and metastasis [17],[18]. High serum levels of miR-19a are also associated with poor outcome in metastatic inflammatory breast cancer [19]. The up-regulation of miR-19a in baldder cancer has been reported by deep sequencing in nine bladder urothelial carcinoma patients [20]. However, the expression pattern and the exact role of miR-19a in bladder cancer have not been elucidated.

In this study, we used Taqman probe stem-loop real-time PCR to accurately measure the levels of miR-19a in 100 pairs of bladder cancer tissues and the adjacent non-neoplastic tissues. We found that miR-19a was significantly up-regulated in bladder cancer tissues. Enforced expression of miR-19a can promote the proliferation of bladder cancer cells, whereas repression of endogenous miR-19a led to the suppression of cell growth of bladder cancer cells. In addition, we improved that miR-19a acted its oncogenic role in bladder cancer partially through targeting PTEN. Furthermore, investigation of miR-19a expression in the plasma of bladder cancer patients showed that miR-19a was also increased in plasma of bladder cancer patients which strongly supported miR-19a could be developed as potential diagnostic marker of bladder cancer.

## Methods

### Cell culture and transfections

The human bladder cancer cell lines (J82, HT1376, RT4, T24 and TCCSUP) and immortalized human bladder epithelium (HCV29 and HU609) cells were propagated in DMEM (Invitrogen) supplemented with 10% FCS at 37°C in 5% CO_2_ cell culture incubator. miR-19a mimics, inhibitors and scramble control were obtained from Dharmacon and transfected with DharmFECT1 (Dharmacon) at a final concentration of 50 nM. The plasmid expressing PTEN was obtained from Origene (SC119965) and co-transfected with miR-19a mimics at 2 μg/ml.

### Patients and specimens

The human clinical samples were collected from surgical specimens from 100 patients with bladder cancer at Suining Central Hospital. The corresponding adjacent non-neoplastic tissues from the macroscopic tumor margin were isolated at the same time and used as controls. All samples were immediately snapped frozen in liquid nitrogen and stored at −80°C until RNA extraction.

Whole blood samples were prospectively collected from bladder cancer patients and control patients without urologic malignancies. Whole blood (5–8 ml) was collected in an ethylene diamine tetracetic acid (EDTA) tube. The sample was centrifuged twice at 4°C. Plasma (supernatant after second centrifugation) was then stored at −80°C. The Clinical Research Ethics Committee of Suining Central Hospital approved the research protocols and written informed consent was obtained from the participants.

### RNA extraction, cDNA synthesis, and real-time PCR assays

Total RNA was extracted from tissues and cells using Trizol reagent (Invitrogen, CA, USA) according to the manufacturer’s instructions. Total RNA of plasma was isolated using a commercially available kit (mirVana; miRNA Isolation Kit, Applied Biosystems, Carlsbad, CA) according to the manufacturer’s protocol. RNA was quantified and cDNA was synthesized by M-MLV reverse transcriptase (Invitrogen) from 2 μg of total RNA. A stem-loop RT primer was used for the reverse transcription. Quantitative RT-PCR was performed in a Bio-Rad CFX96 real-time PCR System (Bio-Rad, CA, USA) using TaqMan probes (Applied Biosystems, Foster City, CA, USA) according to the manufacturer’ s instructions. The PCR conditions were as follows: 95°C for 30 s, followed by 40 cycles of 95°C for 5 s and 60°C for 34 s. The data were normalized using the endogenous U6 snRNA. The 2-ΔΔCT method was used in the analysis of PCR data. Primer sequences are presented in Table 1.

**Table 1:** Sequence of primers used in qRT-PCR

**Primer**	**Sequence (5’ → 3’)**
miR-19a-RT	GTCGTATCCAGTGCAGGGTCCGAGGTATTCGCACTGGATACGACTCAGTTT
miR-19a-forward	CTGGAGTGTGCAAATCTATGC
miR-19a-reverse	GTGCAGGGTCCGAGGT
miR-19a-probe	FAM-CTGGATACGACTCAGTTT-MGB
U6-RT	AAAATATGGAACGCTTCACGAATTTG
U6-forward	CTCGCTTCGGCAGCACATATACT
U6-reverse	ACGCTTCACGAATTTGCGTGTC
U6-probe	FAM-CCATGCTAATCTTCTCTGTA-MGB

### Cell proliferation assay and colony formation assay

To measure the effect of miRNA mimics or FSCN1 siRNA on cell proliferation, cells were incubated in 10% CCK-8 (DOJINDO) diluted in normal culture media at 37°C until visual color conversion appears. Proliferation rates were determined at day 1, 2, 3, 4 post-transfection, and quantification was done on a microtiter plate reader (Spectra Rainbow, Tecan) according to the manufacturer's protocol. Meanwhile, the mimic-transfected cells were trypsinized and replated at 200 cells per well in 6-well plates, cultured for 7 days, then fixed with methanol and stained with 0.1% crystal violet in 20% methanol for 15 min.

### Western blotting

Whole-cell lysate or nuclear extract was subjected to Western blot analysis as described previously [21]. The following antibodies were used for Western blot: GAPDH (10494-1-AP, Proteintech), PTEN (22034-1-AP, Proteintech).

### Statistics

The statistical analyses for miR-19a expression in clinical samples, correlation of miR-19a expression with patients’ clinicopathological variables were conducted using the Bonferroni multiple-comparison test. The other statistical analyses were evaluated by independent samples *T* test (two-tailed). P ≤ 0.05 was considered statistically significant.

## Results

### miR-19a is up-regulated in bladder cancer cells

**Figure 1:**
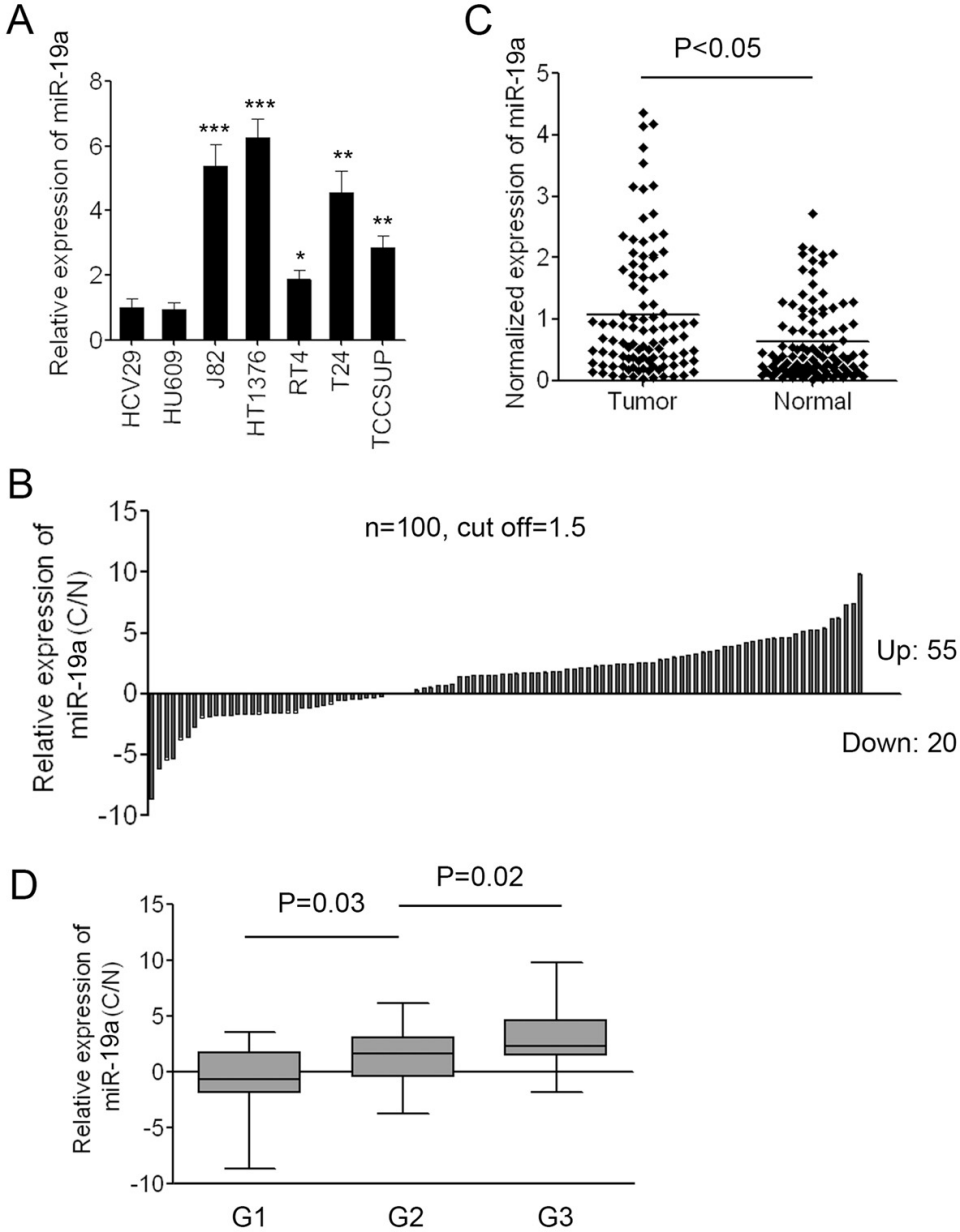
**miR-19a is significantly up-regulated in bladder cancer cell lines and in bladder cancer tissues. (A)** The expression level of miR-19a in two immortalized human bladder epithelium cells (HCV29 and HU609) and five bladder cancer cell lines (J82, HT1376, RT4, T24 and TCCSUP). Data are shown as mean + s.d. (n = 3); * indicates P-value < 0.05; ** indicates P-value < 0.01; *** indicates P-value < 0.001. **(B)** The relative expression of miR-19a in 100 pairs of bladder cancer **(C)** and adjacent non-neoplastic tissues (N). **(C)** Normalized expression of miR-19a in 100 pairs of bladder cancer and adjacent normal tissues. **(D)** The correlation of miR-19a expression with tumor grades of bladder cancer tissues.

To analyze the expression of miR-19a in bladder cancer, q-PCR using Taqman probes was conducted to measure the levels of miR-19a. We firstly examined the expression of mature miR-19a in immortalized human bladder epithelium (HCV29 and HU609) cells and five human bladder cancer cell lines (J82, HT1376, RT4, T24 and TCCSUP). The expression level of miR-19a in bladder cancer cell lines was significant higher than that in the normal bladder epithelium cells. Expression level of miR-19a in RT4 was a little lower than that in the four other bladder cancer cell lines (Figure 1A). These data demonstrated that the up-regulation of miR-19a might be relevant to the genesis and development of bladder cancer.

### miR-19a is up-regulated in bladder cancer tissues compared with the corresponding adjacent non- neoplastic tissues

To further analyze the expression of miR-19a in patients with bladder cancer, we measured the levels of miR-19a in 100 pairs of bladder cancer tissues (C) and the adjacent non-neoplastic tissues (N). The results of PCR showed that 55/100 (55%) of cases had increased levels of miR-19a in bladder cancer tissues compared with the corresponding non-neoplastic tissues when the cutoff was set up as 1.5 (Figure 1B). There were 20/100 (20%) of cases had reduced levels of miR-19a in bladder cancer tissues compared with the adjacent non-neoplastic tissues, 25/100 (25%) of cases in whom the expression of miR-19a was slightly changed in bladder cancer tissues. The results also showed that the average expression of miR-19a in bladder cancer samples was significantly higher than that in the adjacent non-neoplastic tissues (p < 0.05) (Figure 1C).

To further investigate the correlation between the expression of miR-19a and the clinicopathological characteristics, the relative expression of miR-19a in 100 pairs of bladder cancer tissues and adjacent normal tissues were statistically analyzed. The clinicopathological features of bladder cancer patients were summarized in Table 2. Correlation analysis showed that high-level expression of miR-19a in bladder cancer was significantly associated with a more aggressive tumor phenotype (Figure 1D). The data also demonstrated that the expression level of miR-19a had no correlation with age, gender and histological type. Collectively, the data indicated that miR-19a was significantly up-regulated in tumor tissues and might play important roles in bladder carcinogenesis as an oncogenic miRNA.

**Table 2:** Clinicopathological features of bladder cancer patients

**Variables**	**Patients, n**	
	**Total**	**Higher miR-19a**
	**(n = 100)**	**(n = 55)**
**Histology**		
TCC	83	32
TCC with aberrant differentiation	17	23
**Gender**		
Male	75	39
Female	25	16
**Age**		
≥60	62	37
<60	38	18
**Stage**		
Ta	34	15
T1	25	11
T2	18	12
T3	13	10
T4	10	7
**Grade**		
1	25	7
2	40	19
3	35	29
**Progression**		
Yes	33	20
No	67	35

### Enforced expression of miR-19a promotes bladder cancer cell growth and colony formation

**Figure 2:**
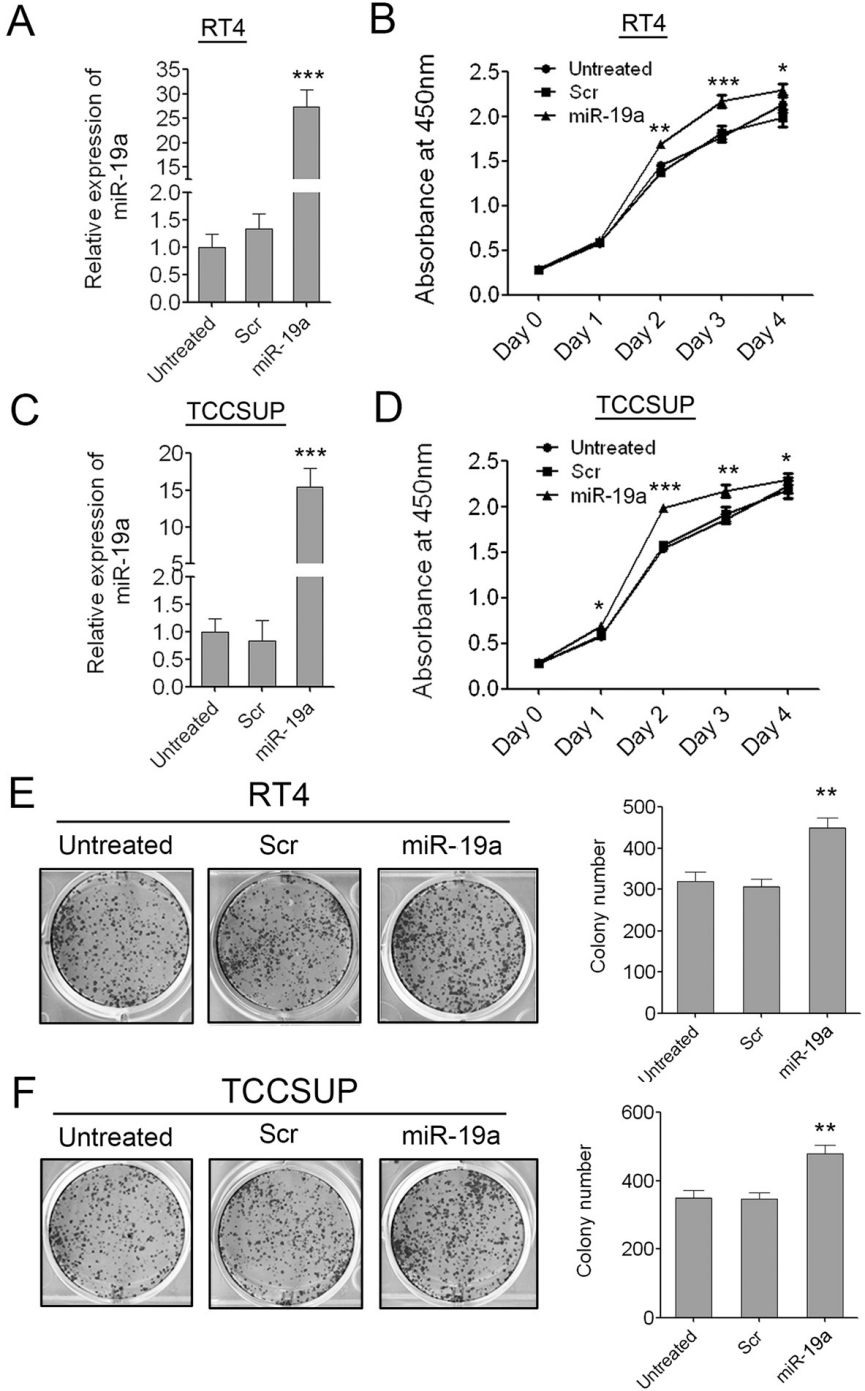
**Enforced expression of miR-19a promotes bladder cancer cell growth and colony formation. (A)** Overexpression of miR-19a in RT4 cells was confirmed by qRT-PCR. **(B)** The cell growth of RT4 cells at 0, 1, 2, 3, 4 days post transfection which was detected by CCK-8 assay. **(C)** Overexpression of miR-19a in TCCSUP cells was confirmed by qRT-PCR. **(D)** The cell growth of TCCSUP cells at 0, 1, 2, 3, 4 days post transfection which was detected by CCK-8 assay. **(E)** The colony number of RT4 cells per well in 6-well plates cultured for 7 days. **(F)** The colony number of TCCSUP cells per well in 6-well plates cultured for 7 days. Data are shown as mean + s.d. (n = 3); * indicates P-value < 0.05; ** indicates P-value < 0.01; *** indicates P-value < 0.001.

To investigate the role of miR-19a in bladder carcinogenesis, we overexpressed miR-19a in the two bladder cancer cell lines RT4 and TCCSUP which had lower expression of miR-19a than the other bladder cancer cell lines. Successful overexpression of miR-19a in the two bladder cancer cell lines was confirmed by q-PCR. miR-19a was overexpressed about 28 folds and 15 folds than the scramble control or untreated RT4 and TCCSUP cells respectively (Figure 2A, C). Consistent with its up-regulation in bladder cancer, the overexpression of miR-19a in both of the two cell lines can promote bladder cancer cell proliferation significantly as demonstrated by CCK-8 assay. The scramble control had no effect on cell proliferation compared with the untreated cells (Figure 2B, D). We also detected the effect of miR-19a on the colony formation ability of bladder cancer cells. The mimic-transfected cells were replated at low density and maintained for 7 days. The overexpression of miR-19a significantly increased the colony number of RT4 and TCCSUP cells, whereas the scramble control had little effect on the colony number compared with the untreated cells (Figure 2E, F). The results proved that miR-19a acted as an oncogenic miRNA in bladder cancer and the up-regulation of miR-19a in bladder tissues would lead to unlimited cell proliferation.

### Attenuated expression of miR-19a in bladder cancer cells can inhibit cell growth and colony formation

**Figure 3:**
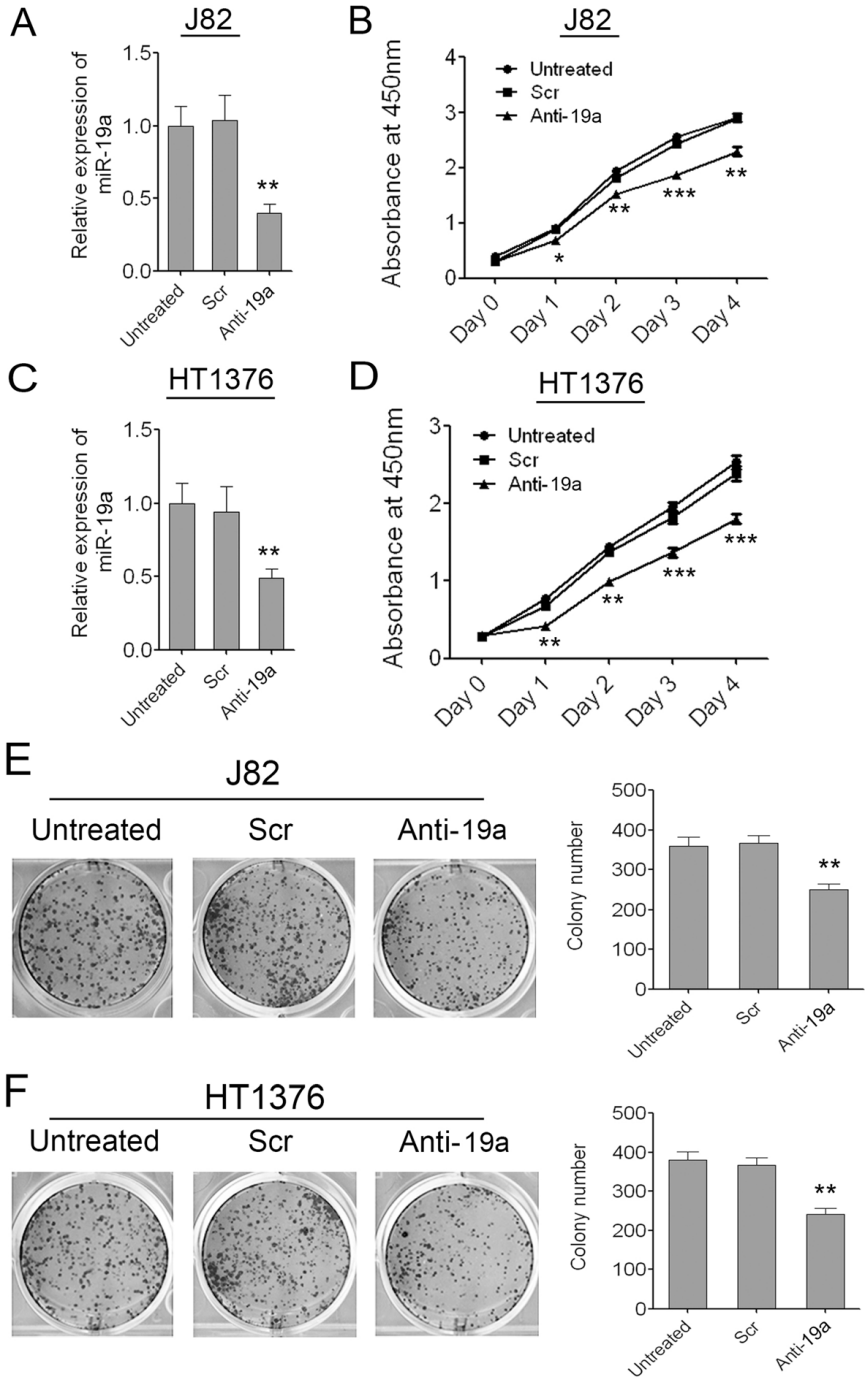
**Attenuated expression of miR-19a in bladder cancer cells can inhibit cell growth and colony formation. (A)** Repression of miR-19a in J82 cells was confirmed by qRT-PCR. **(B)** The cell growth of J82 cells at 0, 1, 2, 3, 4 days post transfection which was detected by CCK-8 assay. **(C)** Repression of miR-19a in HT1376 cells was confirmed by qRT-PCR. **(D)** The cell growth of HT1376 cells at 0, 1, 2, 3, 4 days post transfection which was detected by CCK-8 assay. **(E)** The colony number of J82 cells per well in 6-well plates cultured for 7 days. **(F)** The colony number of HT1376 cells per well in 6-well plates cultured for 7 days. Data are shown as mean + s.d. (n = 3); * indicates P-value < 0.05; ** indicates P-value < 0.01; *** indicates P-value < 0.001.

To further confirm the oncogenic role of miR-19a in bladder carcinogenesis, we suppressed the expression of miR-19a in the two bladder cancer cell lines J82 and HT1376 which had higher expression of miR-19a than the other bladder cancer cell lines. Successful repression of miR-19a in the two bladder cancer cell lines was confirmed by q-PCR (Figure 3A, C). As demonstrated by CCK-8 growth assays, repression of miR-19a reduced cell proliferation in both the two cell lines, whereas the scramble control had no effect on cell proliferation compared with the untreated cells (Figure 3B, D). As demonstrated by the colony formation assay, repression of miR-19a also significantly decreased the colony number of J82 and HT1376 cells, whereas the scramble control had little effect on the colony number compared with the untreated cells (Figure 3E, F). The results proved that miR-19a might act as an oncogenic miRNA in bladder cancer again.

### miR-19a plays its oncogenic role in bladder cancer through targeting PTEN

**Figure 4:**
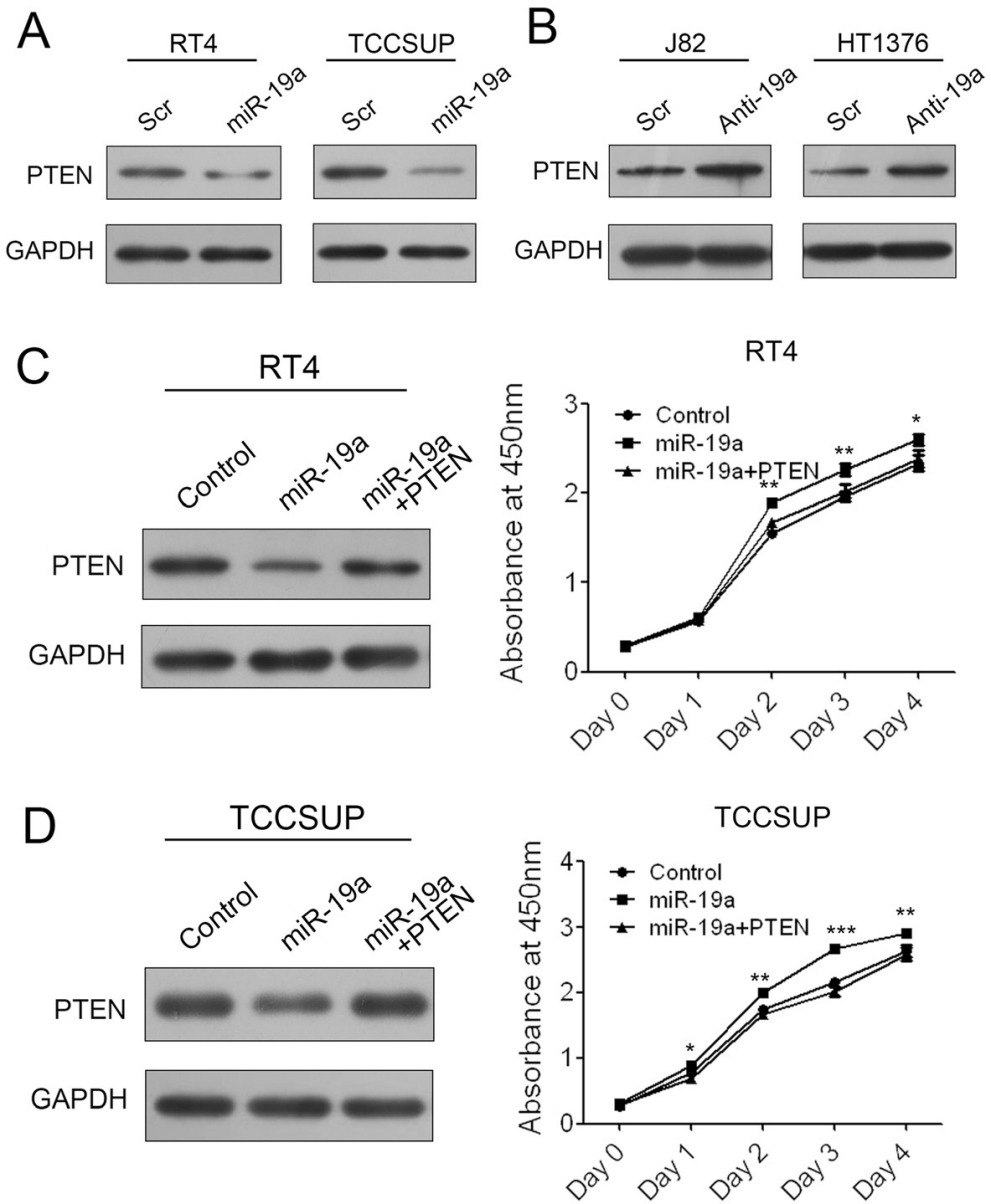
**miR-19a plays its oncogenic role in bladder cancer through targeting PTEN. (A)** Western blot analysis of PTEN expression in RT4 and TCCSUP cells transfected with scramble control or miR-19a mimics. **(B)** Western blot analysis of PTEN expression in J82 and HT1376 cells transfected with scramble control or miR-19a inhibitors. **(C)** Western blot of PTEN expression and CCK-8 analysis of cell growth of RT4 cells transfected with miR-19a mimic and PTEN expression plasmid. **(D)** Western blot of PTEN expression and CCK-8 analysis of cell growth of TCCSUP cells transfected with miR-19a mimic and PTEN expression plasmid.

We further dissected the mechanism of miR-19a functioning as an oncogenic miRNA in bladder cancer. PTEN has been verified as a functional target of miR-19a/b in regulating multidrug resistance in gastric cancer and breast cancer. PTEN acts as a tumor suppressor gene through its phosphatase protein product in a variety of cancers. However, it was still unknown whether miR-19a played its oncogenic roles through targeting PTEN in bladder cancer. So we detected the PTEN protein level in RT4 and TCCSUP cells transfected with miR-19a mimics and also in J82 and HT1376 cells transfected with miR-19a inhibitors. As expected, the PTEN protein level was decreased evidently in presence of miR-19a mimics compared to scramble control in both of RT4 and TCCSUP cells. Conversely, PTEN was increased in presence of miR-19a inhibitors compared to scramble control in both of J82 and HT1376 cells (Figure 4A, B). These results indicated that miR-19a down-regulated PTEN protein in bladder cancer cells.

To further investigate whether miR-19a functions through targeting PTEN in bladder cancer cells, we employed a rescue experiment with miR-19a mimics and PTEN expression plasmid in RT4 and TCCSUP cells. A decrease in PTEN after treatment with miR-19a mimics confirmed the regulatory role of miR-19a on the expression of the target. The addition of PTEN expression plasmid led to further up-regulation of PTEN based on the previously described down-regulation in both of RT4 and TCCSUP cells (Figure 4C, D). Consistent with the restored expression of PTEN protein, promotion of cell growth by miR-19a mimics was rescued by the addition of PTEN expression plasmid (Figure 4C, D). These data confirmed the regulatory role of miR-19a in bladder cancer cells was through targeting PTEN.

### miR-19a is also up-regulated in the plasma of patients with bladder cancer

**Figure 5:**
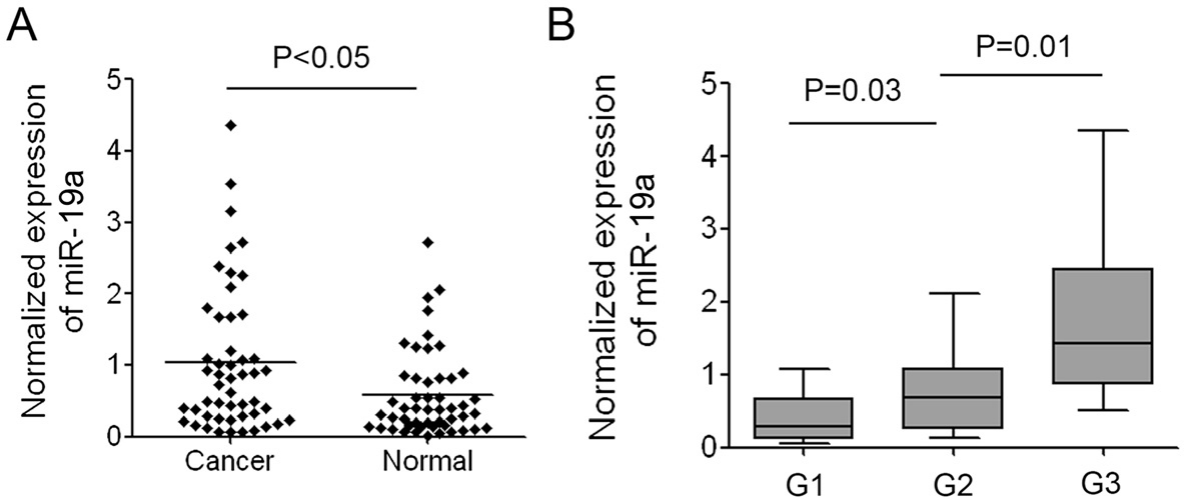
**Expression of miR-19a in the plasma of patients with bladder cancer. (A)** Normalized expression of miR-19a in the plasma of 50 patients with bladder cancer and 50 healthy individuals. **(B)** Correlation of miR-19a expression in the plasma with the tumor grades of bladder cancer.

To explore the diagnostic potential of miR-19a in bladder cancer, we detected the expression of miR-19a in the plasma of 50 patients with bladder cancer and 50 healthy individuals. The data demonstrated that the average level of miR-19a in the bladder cancer patients was significantly higher than that in the healthy individuals which was consistent with its up-regulation in bladder cancer tissues (Figure 5A). The results suggested that miR-19a could be released from the bladder epithelium to the blood and increased miR-19a in the bladder cancer tissues caused its up-regulation in the plasma. The high-level expression of miR-19a in the plasma of bladder cancer patients was also significantly associated with a more aggressive tumor phenotype (Figure 5B). The increased expression of miR-19a in the plasma of bladder cancer patients suggested that miR-19a can be developed as a potential diagnostic marker which can be combined with other miRNAs’ expression to detect bladder cancer.

## Discussion

The up-regulated expression of miR-17–92 cluster has been reported in a variety of cancers including multiple myeloma, leukemia, colorectal cancer and breast cancer [22]–[24]. The miRNA cluster produces a single primary transcript yielding the six mature miRNAs: miR-17, miR-18a, miR-19a, miR-20a, miR-19b, and miR-92a. miR-19 has been identified as the key member responsible for the oncogenic activity [25],[26]. However, the role of miR-19 in bladder cancer remains unknown. In this study, we investigated the expression of miR-19a in a great deal of patients with bladder cancer and dissected the roles and mechanisms of miR-19a in bladder cancer carcinogenesis. We found that miR-19a was significantly up-regulated in bladder cancer tissues and the high expression of miR-19a was associated with the more aggressive phenotypes of bladder cancer. Gain or loss of function of miR-19a in bladder cancer cells also indicated that miR-19a can promote cell growth which was consistent with its role in other cancer types. The important role of miR-19a in regulating bladder cancer cell invasion, migration and *in vivo* carcinogenesis needs to be further confirmed. In case anti-miRs of miR-19a can suppress tumor growth *in vivo* significantly, miR-19a can be further developed as new target for bladder cancer therapy as miRNAs has advantages of being small and easy to delivery, safer than other gene therapy methods [27],[28].

To further dissect the mechanism by which miR-19a functioned as an oncogenic miRNA in bladder cancer, we analyzed the relationship of miR-19a and PTEN in bladder cancer and found that the regulatory role of miR-19a in bladder cancer cells was dependent on targeting PTEN. PTEN is identified as a tumor suppressor that is mutated in a large number of cancers at high frequency. It negatively regulates intracellular levels of phosphatidylinositol-3,4,5-trisphosphate in cells and functions as a tumor suppressor by negatively regulating AKT/PKB signaling pathway [29]–[31]. AKT/PKB signaling pathways answer to growth factors and other extracellular stimuli to regulate several cellular functions including nutrient metabolism, cell growth, apoptosis and survival. miR-19a may repress the expression of PTEN which further lead to the unlimited cell proliferation of bladder cancer cells. The correlation of expression level of miR-19a and PTEN in patients with bladder cancer will be investigated further to confirm that PTEN was a direct target of miR-19a in bladder cancer.

Earlier studies discovered that extracellular miRNAs circulated in the bloodstream and the circulating miRNAs were remarkably stable. Detection of elevated levels of tumor associated miRNAs in serum of patients with diffuse large B-cell lymphoma [32] leads to widely investigation of circulating miRNAs in many human cancers, including breast cancer [33], lung cancer [34], prostate cancer [35], and renal cell carcinoma [36] and so on. The expression profile of miRNAs in serum/plasma of the patients with bladder cancer was also investigated and some important circulating miRNAs in bladder cancer had been identified [37],[38]. These studies support the use of serum/plasma miRNAs as noninvasive means of bladder cancer detection. Serum miR-19a expression has been reported to correlate with worse prognosis of patients with non-small cell lung cancer [39]. We detected the level of miR-19a in plasma of patients with bladder cancer and found that miR-19a was also increased which was consistent with its high level in the cancer tissues. The up-regulation of miR-19a in the plasma might origin from the tumor cells which needs to be improved further. MiRNAs can be detected easily in small amount samples and are stable against degradation and can be detectable in bodily fluids including serum, plasma, saliva, urine and tears [40],[41]. The innate properties of miRNAs make them attractive as potential biomarkers. So miR-19a can be developed as a new diagnostic marker for bladder cancer detection. Further analysis of the correlation of miR-19a expression level with clinical outcome will offer important information about the relationship of miR-19a levels with the clinical diagnosis, therapy and outcome, which will be useful for individualized therapies. In consideration of the possible secretion of miR-19a from the tumor cells to the plasma, the level of miR-19a in urine samples of the patients will be examined. Voided urine can be noninvasively obtained, be designed not only for diagnosis, but also for monitoring disease recurrence and response to therapy [42],[43]. So development of miR-19a as a novel urinary biomarker for bladder cancer will be urgently required for early detection of cancer and individualized therapies.

## Conclusion

In summary, we determined the high expression of miR-19a in the cancer tissues and plasma of patients with bladder cancer and also indicated the oncogenic roles of miR19a in bladder cancer which was dependent on targeting PTEN. Our data provided the potential diagnostic and therapeutic roles of miR-19a in bladder cancer firstly.

## Competing interests

The authors declare no competing financial interests.

## Authors’ contributions

Y-GF conceived the project; designed the experiments and carried out the majority of the experiments; JL conducted the bioinformatics analysis; Y-MK, YH and BL helped to collect clinical samples. PY and ZY helped to culture cells; all authors discussed the results; Y-GF and JL wrote the manuscript. All authors read and approved the final manuscript.
